# Research on the driving mechanism of tourists’ ecological protection behavior in intangible cultural heritage sites

**DOI:** 10.3389/fpsyg.2024.1514482

**Published:** 2024-12-19

**Authors:** Wei Zhang, Hao Ran

**Affiliations:** School of Economics and Management, Yunnan Minzu University, Kunming, Yunnan, China

**Keywords:** intangible cultural heritage sites, Theory of Planned Behavior, ecological protection behavior, tourists, altruism

## Abstract

Despite the increasing focus on intangible cultural heritage tourism, there is a lack of research on the ecological protection behaviors of tourists in these contexts. With UNESCO’s continuous refinement of the World Heritage system, intangible cultural heritage has gradually become a focal point for tourism development and protection. While such tourism can promote the preservation and transmission of heritage, it also introduces ecological environmental issues that need to be addressed. Therefore, exploring the driving mechanisms of tourists’ ecological protection behavior holds significant practical value. Based on the Theory of Planned Behavior (TPB), this study constructs a driving model of tourists’ ecological protection behavior. It examines the influence of behavioral attitude, subjective norms, perceived behavioral control, and personal norms on tourists’ willingness to engage in ecological protection. By distributing questionnaires both offline and online, we analyzed data from 312 valid responses. The results indicate that all four factors have a significant positive impact on tourists’ willingness to engage in ecological protection behavior. Among these factors, personal norms and behavioral attitude have a relatively larger influence. The findings provide valuable references for intangible cultural heritage sites in China and regions with similar cultural and tourism dynamics.

## Introduction

With UNESCO’s continuous improvement of the World Heritage system, intangible cultural heritage has been incorporated into the realm of world heritage, becoming an increasingly hot topic in both academia and practice (Tavares et al., 2021; Yuan et al., 2022). In this context, promoting the protection and inheritance of intangible cultural heritage through tourism development has become a focal point of widespread concern. The rapid development of the tourism industry has made this approach not only significant in practice but also an inevitable trend (Radosavljević and Kuletin Ćulafić, 2019). However, while intangible cultural heritage tourism promotes preservation and transmission, it also introduces ecological environmental issues that need to be addressed. Therefore, researching how to effectively integrate intangible cultural heritage into tourist attractions and carrying out scientific planning and development to resolve potential conflicts is an urgent topic that requires in-depth exploration.

As a tourism resource, intangible cultural heritage undoubtedly has feasibility (Maldonado-Erazo et al., 2023). However, research on its sustainable development remains insufficient (Tan et al., 2018; Walker et al., 2021). It is undeniable that the tourism development of intangible cultural heritage sites may bring some negative impacts, especially the destruction of the original environment of the intangible cultural heritage. For instance, the influx of tourists can lead to environmental degradation in heritage sites, highlighting the need for effective ecological protection measures. During the development of intangible cultural heritage tourism resources, only by protecting the ecological environment of heritage sites can we ensure the effective protection and transmission of intangible cultural heritage, thereby achieving positive development (Zhang, 2020). The ecological protection of intangible cultural heritage sites not only means protecting the intangible cultural heritage itself but also maintaining the ecological environment on which it depends. Ecological protection provides a safety barrier for intangible cultural heritage, allowing it to be preserved completely in its original environment, thus becoming a “complete culture.” The authenticity and integrity of this culture have a stronger appeal to tourists. However, balancing tourists’ needs for cultural experiences with the responsibility of protecting intangible cultural heritage poses significant challenges.

Moreover, the conscious participation of the public is the lasting driving force for the protection of intangible cultural heritage. Public integration and participation are important guarantees for enhancing social cohesion and promoting civic vitality. This concept has become a consensus in the protection of intangible cultural heritage. Awakening the public’s sense of responsibility and mission is crucial for the protection of intangible cultural heritage. Therefore, exploring the driving mechanisms of public ecological protection is an important aspect of promoting the sustainable development of intangible cultural heritage tourism.

## Literature review

Intangible cultural heritage is considered the core spirit of a cultural and social group (Zhang et al., 2019). Specifically, it is embodied as “culture practiced by people in daily life,” including beliefs, viewpoints, ephemeral performances, and events, rather than tangible cultural objects such as monuments or artifacts. Intangible cultural heritage is not only an important symbol of cultural identity but also a crucial tool for promoting social cohesion and advancing peace processes. In regions with sharp social conflicts, intangible cultural heritage has been proven to play a key role in post-conflict reconciliation and reconstruction (Bräuchler, 2011).

In China, stakeholders in intangible cultural heritage tourism mainly include inheritors, the government, communities, developers, tourists, experts, and non-governmental organizations. The protection of intangible cultural heritage and tourism development should form an organic comprehensive system, with inheritors at the core. The government provides support through laws and policies, developers are responsible for capital investment, communities actively participate, and tourists voluntarily maintain this cultural heritage. The government plays an irreplaceable leading role by promoting the implementation of protection work through policy guidance and resource allocation. Meanwhile, communities and inheritors, as the core forces of intangible heritage protection, bear the direct responsibility of cultural transmission (Gao et al., 2022; Zhao et al., 2022). The participation of communities and residents is the foundation of the sustainability of protection work (Wei et al., 2021; Maldonado-Erazo et al., 2023). Studies have explored the relationship between heritage protection and tourism development from multiple dimensions such as government actions, management strategies, and community participation (Aas et al., 2005; Rasoolimanesh et al., 2017). Additionally, experts and non-governmental organizations have also played important roles in the development of intangible cultural heritage tourism. These entities not only provide scientific support through research and monitoring but also assume supervisory functions to some extent. They effectively curb over-commercialization and vulgarization, thus ensuring a balance between heritage protection and tourism development. In summary, the coordinated cooperation of multiple stakeholders is key to achieving the sustainable protection and development of intangible cultural heritage.

However, in addition to these stakeholders, tourists themselves play an indispensable role in cultural experience and dissemination (Palau-Saumell et al., 2013). On one hand, intangible cultural heritage provides tourists with immersive cultural experiences through its unique cultural charm (Yan et al., 2024). By participating in traditional ceremonies, learning handicrafts, or watching performing arts, tourists can not only feel the uniqueness of local culture but also enhance their identification and emotional connection with intangible cultural heritage (Su et al., 2020; Lan et al., 2021; Chen, 2021). This cultural experience satisfies tourists’ spiritual needs while also promoting the social dissemination of intangible heritage, allowing it to transcend local boundaries and gain broader recognition and support. On the other hand, tourists’ behaviors have a profound impact on the protection and transmission of intangible cultural heritage sites. Tourists’ positive consumption behaviors, such as purchasing local specialty products or participating in cultural activities, not only bring direct economic benefits to inheritors and communities but also provide sustainable financial support for heritage protection (Guo and Zhu, 2023; Qiu and Zuo, 2023). However, tourists’ behaviors during cultural experiences may also pose potential risks. For example, due to insufficient understanding of cultural connotations, some tourists may engage in inappropriate behaviors, such as disrupting traditional ceremonies or misinterpreting forms of intangible heritage expression. These actions can weaken the cultural authenticity and sacredness of the heritage. To balance tourists’ needs for cultural experiences with the responsibility of protecting intangible cultural heritage, the key lies in enhancing tourists’ cultural awareness and behavior norms through education and guidance. For instance, conducting interactive cultural experience programs and setting up educational guided tours at heritage sites can help tourists more comprehensively understand the cultural value and ecological significance of intangible heritage. This understanding can reduce destructive behaviors and promote responsible cultural consumption (Ott et al., 2015; Yan and Li, 2023). In this way, tourists are not only experiencers of cultural values but also important driving forces for the protection of intangible heritage.

Several related studies on heritage tourism have adopted the tourists’ perspective. For example, Williams and Soutar (2009), Prebensen et al. (2012), Wu and Li (2014), and Chen et al. (2016) examined the relationship between service quality, perceived tourism value, environmental behavior, and heritage protection from the tourists’ viewpoint. Additionally, Hungerford and Volk (1990), Azman et al. (2010), and Palau-Saumell et al. (2013) focused on tourists’ knowledge of heritage protection and related education. They suggested that enhancing tourists’ awareness of protection can encourage them to adopt more responsible environmental behaviors. These studies from the tourists’ perspective provide important references for the sustainable development of tourism and heritage protection.

However, although the role of tourists in heritage tourism has gradually received attention, existing research mainly focuses on tangible cultural heritage sites. In contrast, systematic discussions on how tourists can effectively participate in intangible cultural heritage tourism and support the sustainable development of intangible cultural heritage sites through behavioral changes are still insufficient. Buckley (2017) argued that although tourism companies and merchants may actively promote the protection of heritage sites, there is a lack of empirical support for tourists’ autonomous participation.Based on this, this paper takes the tourists’ perspective as the entry point to explore key issues related to intangible cultural heritage site tourism, aiming to supplement existing research. This study intends to further deepen the understanding of the driving mechanisms of tourists’ behavior and provide practical references for intangible cultural heritage site managers and policymakers to promote sustainable tourism development.

## Research model and hypotheses

The Theory of Planned Behavior posits that behavioral decisions are mainly determined by individual volitional factors. That is, an individual’s intention to engage in a certain behavior is the main determinant of their actual behavior (Ajzen, 1991). The core assumption of the theory is that individuals tend to choose behavioral options that bring the greatest benefits, the lowest costs, or the minimal negative impacts. In this model, attitude, subjective norms, and perceived behavioral control are considered to have a positive influence on behavioral intention. This has been confirmed by many studies (Chan and Bishop, 2013; Han, 2015). When an individual has a more positive attitude, receives more support from others, and has stronger perceived behavioral control, their behavioral intention is greater. Additionally, other empirical studies have shown that the Theory of Planned Behavior is widely applicable in explaining various behaviors in the fields of environmental protection, tourism, and hospitality (Mancha and Yoder, 2015; Han, 2015; Shin et al., 2018; Wang et al., 2020; Lee et al., 2021).

However, although the TPB has high applicability in explaining behavioral intentions, in the field of ecological protection behavior, the relative influence of attitude, subjective norms, and perceived behavioral control on behavioral intentions may vary due to situational factors. Moreover, the model has been criticized by many researchers for its weak explanatory assumptions (Juvan and Dolničar, 2016). For example, Hughes (2013) pointed out that in studying Australian families participating in wildlife viewing experiences, tourists’ intentions may not be sufficiently reliable in predicting changes in tourism behavior, especially in the context of long-term behavioral changes. Therefore, there is a need to explore its influencing mechanisms in the fields of ecological protection behavior and cultural heritage within the existing framework. This paper attempts to provide new empirical evidence for the optimization and expansion of the TPB model and further extend its application context. Specifically, we propose that the three antecedent variables of the TPB—behavioral attitude, subjective norms, and perceived behavioral control—are applicable in influencing behavioral intentions in the context of tourists’ ecological protection behavior decisions at intangible cultural heritage sites and can be further verified. Accordingly, this study proposes the following research hypotheses:

*Hypothesis 1*: Behavioral attitude has a significant positive impact on tourists’ willingness to engage in ecological protection behavior at intangible cultural heritage sites.

*Hypothesis 2*: Subjective norms have a significant positive impact on tourists’ willingness to engage in ecological protection behavior at intangible cultural heritage sites.

*Hypothesis 3*: Perceived behavioral control has a significant positive impact on tourists’ willingness to engage in ecological protection behavior at intangible cultural heritage sites.

Since the 1970s, the concept of “altruism” has attracted increasing attention from scholars (Schwartz, 1977; Fennell, 2006). Altruistic behavior emphasizes higher moral standards and obligations. This concept refers to situations where individuals are willing to sacrifice their own interests to benefit others. In this process, personal norms, as an internalized moral belief, play a core driving role. The concept of altruism has been widely used to explain prosocial behaviors related to environmental and sustainability issues (Jouvet et al., 2000; Teng et al., 2013; Daube and Ulph, 2014; Gao et al., 2016; Hanafiah, 2022; Shrum et al., 2022). This paper further incorporates personal norms as a moral driving factor into the framework of the TPB to explore its role mechanism in ecological protection behavioral intentions. By introducing personal norms, this research not only deepens the understanding of the TPB but also reveals the key significance of altruism and personal norms in ecological protection and cultural contexts. It thereby provides empirical support for incorporating moral factors into predictive models. We proposes that integrating personal norms into the framework of the TPB can significantly enhance the explanatory power of ecological protection behavioral intentions. Specifically, personal norms, as an internal moral driving factor, work together with attitude, subjective norms, and perceived behavioral control to construct a more comprehensive mechanism to systematically analyze the formation process of behavioral intentions. Based on the above analysis, this study further proposes the following hypothesis:

*Hypothesis 4*: Personal norms have a significant positive impact on tourists’ willingness to engage in ecological protection behavior at intangible cultural heritage sites.

**Figure 1:**
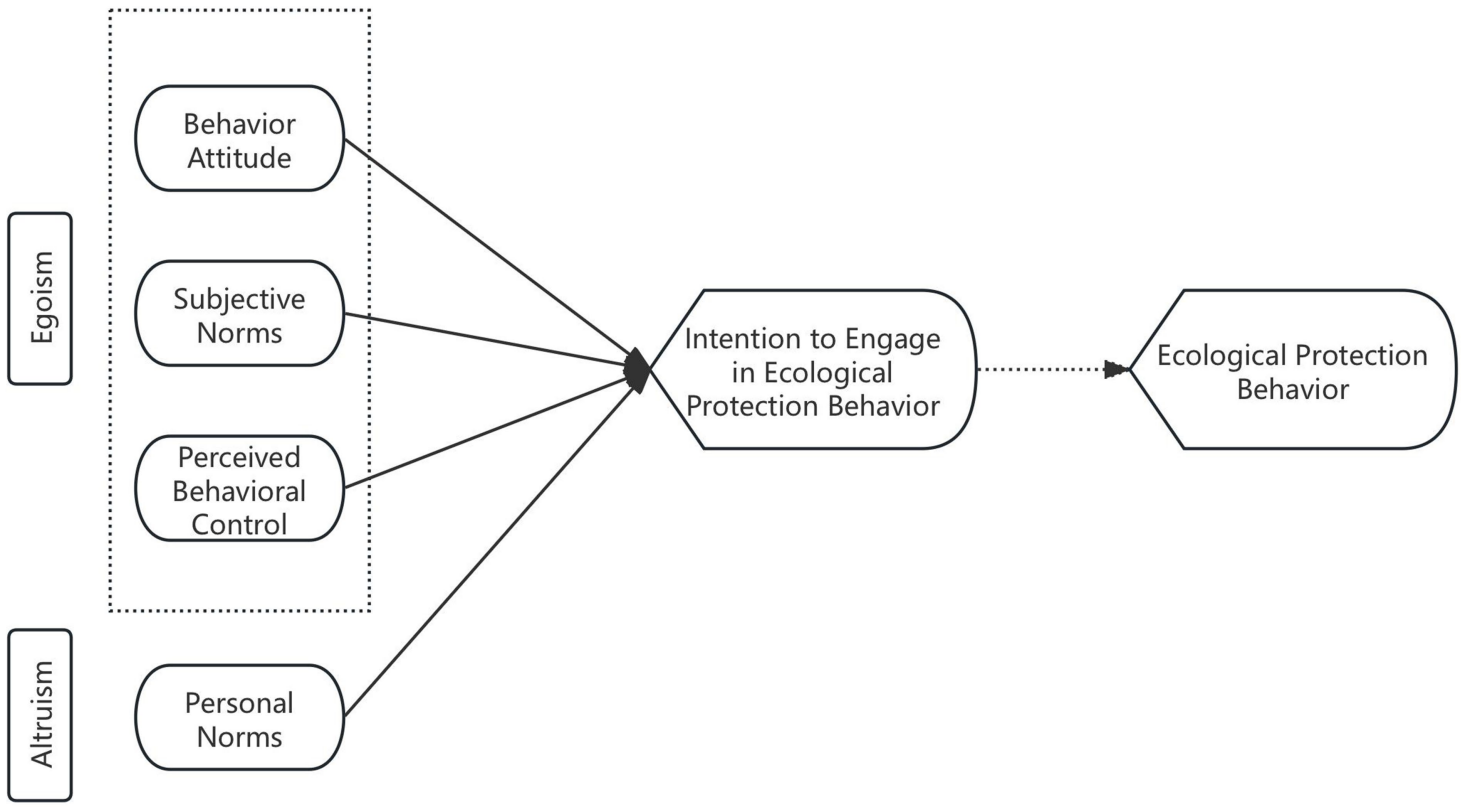
Model of the driving mechanism of tourists’ ecological protection behavior in intangible cultural heritage sites. Solid lines represent the paths to be verified in this paper, while dashed lines indicate paths confirmed by existing theories.

Combining the above hypotheses, the research model of this paper is shown in Figure 1.

## Research design

### Variable measurement

The research variables in this paper include behavioral attitude, subjective norms, perceived behavioral control, personal norms, and intention. Considering the scientificity and accuracy of measurement, the measurement of variables in this study refers to the research of multiple scholars and is modified according to the context of this paper. Specifically: The items for behavioral attitude and subjective norms refer to the studies of Ajzen (1991) and Qiu (2017), each containing three items. These items were adapted to reflect the context of intangible cultural heritage sites by focusing on specific behaviors relevant to these settings. Perceived behavioral control refers to the scale of Song et al. (2014), involving three items. The personal norms scale refers to the research of Esfandiar et al. (2019), with three items. Intention refers to the study of Chen and Tung (2014), containing three items. The variable measurement items are shown in Table 1. The questionnaire uses a five-point Likert scale, where 1 represents “strongly disagree/very inconsistent” and 5 represents “strongly agree/very consistent.” The higher the number, the greater the degree of agreement or consistency.

**Table 1:** Variable measurement items.

Variable	Measurement items	References
Behavioral attitude (AT)	AT1: My ecological protection behavior at intangible cultural heritage sites is necessary. AT2: My ecological protection behavior at intangible cultural heritage sites is meaningful. AT3: My ecological protection behavior at intangible cultural heritage sites is valuable.	Ajzen (1991) and Qiu (2017)
Subjective norms (SN)	SN1: People important to me support my ecological protection of intangible cultural heritage sites. SN2: People important to me think I should engage in ecological protection at intangible cultural heritage sites. SN3: People important to me suggest I engage in ecological protection at intangible cultural heritage sites.	Ajzen (1991) and Qiu (2017)
Perceived behavioral control (PBC)	PBC1: As long as I am willing, I can easily engage in ecological protection at intangible cultural heritage sites. PBC2: As long as I am willing, I am fully capable of engaging in ecological protection at intangible cultural heritage sites. PBC3: As long as I am willing, I have enough time to engage in ecological protection at intangible cultural heritage sites.	Song et al. (2014)
Personal norms (PN)	PN1: At intangible cultural heritage sites, I have an obligation to protect the ecology. PN2: When visiting intangible cultural heritage sites, I have a responsibility to protect the ecology. PN3: If I do not protect the ecology when visiting intangible cultural heritage sites, I would feel guilty.	Esfandiar et al. (2019)
Intention (BI)	BI1: At intangible cultural heritage sites, I would sacrifice my comfort to engage in ecological protection. BI2: At intangible cultural heritage sites, I would sacrifice my time to engage in ecological protection. BI3: At intangible cultural heritage sites, I would put ecological protection into action.	Chen and Tung (2014)

### Data collection and sample description

After defining the measurement instruments, data were collected to test the hypotheses. The questionnaire was designed using the Questionnaire Star platform and data were collected through a combination of online and offline methods.

The offline survey was conducted in the Ancient City of Dali, Yunnan. This city is not only an important birthplace of Bai culture but also an essential window for understanding Dali’s intangible cultural heritage. The Ancient City of Dali attracts a large number of tourists with its rich intangible cultural heritage resources. Tourists can experience tie-dyeing techniques, appreciate traditional Bai architecture, and participate in cultural activities such as the Bai “Three-Course Tea” performance within the ancient city. On November 29, 2022, the Bai “Three-Course Tea” was inscribed on UNESCO’s Representative List of the Intangible Cultural Heritage of Humanity as a protected item under “Traditional Chinese Tea Processing Techniques and Associated Social Practices.” This international recognition further highlights the important status and cultural value of the Ancient City of Dali in intangible cultural heritage protection. Choosing the Ancient City of Dali as the offline survey location is based on its typicality and exemplarity in the protection and transmission of intangible cultural heritage. Unlike other heritage sites that may focus on tangible artifacts, Dali offers rich intangible cultural experiences, making it ideal for our study. On one hand, the Ancient City of Dali gathers multiple intangible cultural heritage projects, making it an ideal field for studying the relationship between cultural experience and ecological protection behavior. On the other hand, as a well-known international tourism destination, the Ancient City of Dali attracts a wide range of tourist groups, reflecting the behavioral intentions of tourists from different cultural backgrounds toward ecological protection. The online questionnaire was distributed through mainstream social media platforms such as WeChat Moments, Weibo, and QQ to cover a broader audience. It targeted not only tourists who have visited the Ancient City of Dali but also those with experience in other intangible cultural heritage sites or attractions, increasing the diversity and representativeness of the sample. Combining online and offline data collection allowed us to reach a diverse sample, enhancing the generalizability of our findings. This method provides reliable data support for studying intangible cultural heritage tourism and ecological protection behaviors while ensuring cultural diversity and external validity.

A total of 350 questionnaires were distributed in this survey, and 312 valid questionnaires were finally recovered, with an effective recovery rate of 89.14%. Among these 312 valid samples, males accounted for 50.64%, and females accounted for 49.36%. In terms of age distribution, the 26–35 age group had the highest proportion at 24.36%; followed by those over 55 years old at 21.76%; the 36–45 and 46–55 age groups both accounted for 20.83%; the group under 25 years old accounted for 12.18%. In terms of educational level, high school or technical secondary school accounted for the highest proportion at 32.05%; followed by junior college at 28.21%; undergraduate degrees accounted for 19.87%; those with education below junior high school accounted for 13.14%; postgraduate and above accounted for 6.73%. The data shows that 45.83% of respondents had one experience at the target intangible cultural heritage site or attraction, 32.69% had visited 2–3 times, 13.78% had visited 4–5 times, and 7.69% had visited more than 5 times.

## Empirical analysis

### Reliability and validity analysis

SPSS software was used for reliability and factor analysis of the scale. Firstly, Cronbach’s *α* coefficient was used for reliability testing to assess the stability and reliability of the scale. Values above 0.75 indicate good reliability. As shown in Table 2, the Cronbach’s α coefficients for the five variables—behavioral attitude, subjective norms, perceived behavioral control, personal norms, and intention—are 0.885, 0.876, 0.909, 0.878, and 0.898, respectively. All coefficients exceed the acceptable level, indicating high internal consistency and good reliability. Subsequently, validity analysis was conducted based on the factor loadings of each variable. The results show that the composite reliability (CR) is greater than 0.75, and the average variance extracted (AVE) is greater than 0.5, indicating that the scale has good convergent validity. As shown in Table 3, the KMO value is 0.982, and the significance of Bartlett’s test of sphericity is *p* < 0.001. Both metrics meet the standards, indicating that the scale is suitable for further analysis.

**Table 2:** Reliability and validity analysis results.

Variable	Cronbach’s *α*	CR	AVE
Behavioral attitude	0.885	0.885	0.720
Subjective norms	0.876	0.876	0.702
Perceived behavioral control	0.909	0.909	0.768
Personal norms	0.878	0.878	0.706
Intention	0.898	0.899	0.747

**Table 3:** KMO and Bartlett’s test results.

KMO Value	0.982	
Bartlett’s Test	Approx. Chi-Square	4940.389
	DF	105
	P	0.000***

*** indicates significance at the 1% level.

### Hypothesis testing

Descriptive statistics were conducted on the overall sample data. As shown in Table 4, the absolute values of skewness and kurtosis indicate that the data distribution is approximately normal.

**Table 4:** Skewness and kurtosis test results.

Item	Skewness	Kurtosis
AT1	−0.969	−0.039
AT2	−1.028	−0.148
AT3	−0.973	−0.045
SN1	−0.946	−0.213
SN2	−0.957	0.020
SN3	−0.869	−0.211
PBC1	−0.992	−0.058
PBC2	−0.960	−0.008
PBC3	−0.995	0.001
PN1	−0.880	−0.156
PN2	−0.922	−0.017
PN3	−0.812	−0.211
BI1	−0.957	−0.098
BI2	−0.837	−0.269
BI3	−0.995	−0.005

A multiple regression analysis was conducted to explore the relationship between the dependent variable (ecological protection behavioral intention) and the independent variables (behavioral attitude, subjective norms, perceived behavioral control, personal norms). The interpretation of statistical results is crucial for understanding the implications. Table 5 shows that the Durbin-Watson value of the model is 2.057, within the acceptable range of 1.5 to 2.5, indicating that the sample data are free from autocorrelation.

**Table 5:** Model summary.

R	R Square	Adjusted R Square	Std. Error of the Estimate	Durbin-Watson
0.935	0.875	0.873	0.399	2.057

Predictors include behavioral attitude, subjective norms, perceived behavioral control, and personal norms.

To further assess the practical contribution of the predictor variables, Cohen’s $f^{2}$ effect size was calculated. According to the formula $f^{2}=\frac{R^{2}}{1-R^{2}}$ , the effect size was calculated as $f^{2}=\frac{0.871}{1-0.871}=7$ . Based on Cohen (1990) standards, an $f^{2}$ = 7 in this study indicates that the total contribution of the predictor variables has great practical significance, demonstrating that behavioral attitude, subjective norms, perceived behavioral control, and personal norms have very significant predictive power on ecological protection behavioral intention. This result further supports the theoretical framework and variable selection.

As shown in Table 6, the Variance Inflation Factor (VIF) values of the four variables range between 5.742 and 6.852, indicating moderate multicollinearity but not reaching the critical value of severe multicollinearity (VIF ≥ 10). This suggests that the multicollinearity issue of the model is within an acceptable range, and the robustness and explanatory power of the model are not significantly affected. The significance *p*-values of all variables are less than 0.01, indicating that tourists’ behavioral attitude, subjective norms, perceived behavioral control, and personal norms are all significantly positively correlated with ecological protection behavioral intention. Based on the analysis results, the hypothesis testing outcomes are as follows:

**Table 6:** Regression analysis results.

Hypothesis path	Standardized coefficient (β)	*p*-value	*t*-value	VIF	Conclusion
Behavioral Attitude → Ecological Protection Intention	0.280	<0.001***	5.288	6.852	Supported
Subjective Norms → Ecological Protection Intention	0.177	<0.001***	3.660	5.742	Supported
Perceived Behavioral Control → Ecological Protection Intention	0.229	<0.001***	4.388	6.696	Supported
Personal Norms → Ecological Protection Intention	0.295	<0.001***	5.920	6.069	Supported

*** indicates significance at the 1% level.

*Hypothesis 1*: Tourists’ behavioral attitude has a significant positive impact on ecological protection behavioral intention (*β* = 0.280, *p* < 0.001), supporting Hypothesis 1.

*Hypothesis 2*: Subjective norms also have a significant positive impact on ecological protection behavioral intention (*β* = 0.177, *p* < 0.001), confirming Hypothesis 2.

*Hypothesis 3*: Perceived behavioral control shows a significant positive impact (*β* = 0.229, *p* < 0.001), supporting Hypothesis 3.

*Hypothesis 4*: Tourists’ personal norms are significantly positively correlated with ecological protection behavioral intention (*β* = 0.295, *p* < 0.001), proving Hypothesis 4.

These findings provide empirical support for our proposed model, which we discuss in the following section.

## Research conclusion and implications

### Research conclusion

The results indicate that tourists’ behavioral attitude, subjective norms, perceived behavioral control, and personal norms all have significant positive impacts on ecological protection behavioral intention, validating Hypotheses 1 through 4. A β value of 0.295 for personal norms suggests it has the strongest influence on ecological protection intention. This implies that tourists’ internalized moral sense of responsibility plays a core role in promoting ecological protection behaviors, aligning with existing research that emphasizes the importance of personal norms in prosocial behavior (Schwartz, 1977). Behavioral attitude (*β* = 0.280) follows, indicating that tourists’ positive evaluations of ecological protection can significantly enhance their behavioral intentions. Perceived behavioral control (*β* = 0.229) and subjective norms (*β* = 0.177) have relatively weaker influences but remain significant, confirming the TPB’s predictions regarding these variables’ impact on behavioral intention. For instance, unlike studies by Han (2015) and Lee et al. (2021) that emphasize behavioral attitude and subjective norms as primary drivers, our study identifies personal norms as the most influential factor. This suggests that in ecological protection behavior, the relative influence of different variables may vary due to specific contexts and backgrounds.

### Implications

#### Strengthen the internalization and shaping of personal norms

Given that personal norms have the strongest influence on promoting tourists’ ecological protection behavioral intention (*β* = 0.295), individuals’ internal moral sense of responsibility is the core driving force for initiating ecological protection behavior. Therefore, it is necessary to enhance tourists’ recognition of personal norms through various means, internalizing them as the fundamental force driving long-term behavior. Governments and educational departments need to integrate the value concepts of ecological protection into social governance through policy design and education. This could involve promoting the shared values of ecological protection and cultural transmission in cultural festivals, intangible heritage exhibitions, and school curricula. Furthermore, cultural and tourism departments and scenic area managers can strengthen tourists’ emotional connections through experiential activities. For example, organizing tourists to participate in ecological intangible heritage skill protection projects highlights their actual impact on culture and the environment. Communities can host local environmental-themed activities, collaborating with tourists to explore ecological protection implementations at intangible cultural heritage sites. Embedding ecological protection awareness into community culture enhances the public’s moral responsibility.

#### Enhance tourists’ positive attitude toward ecological protection

Behavioral attitude is one of the important factors influencing tourists’ ecological protection behavioral intention (*β* = 0.280). This finding indicates that when tourists have a more positive cognition and evaluation of ecological protection behavior, their willingness to protect will significantly increase. Therefore, relevant parties should focus on enhancing tourists’ positive perception of ecological protection through various forms, enabling them to recognize the value and importance of protective behavior. Cultural and tourism departments can design rich experiential ecological tourism activities, allowing tourists to personally participate in ecological protection practices. For instance, organizing environmental volunteer activities, such as heritage site vegetation restoration or cultural landscape recovery projects, enables tourists to feel the effectiveness and significance of protective behaviors through actual actions. Additionally, media platforms can be utilized to promote successful cases of ecological protection. Visually demonstrating the outcomes of protective behaviors in improving heritage site environments, enhancing cultural value, and creating social benefits further strengthens tourists’ confidence and recognition of ecological protection.

#### Enhance tourists’ perceived behavioral control over ecological protection actions

Perceived behavioral control—that is, tourists’ belief in whether they have the ability to implement ecological protection behavior (*β* = 0.229)—significantly affects their behavioral intention. To reduce tourists’ perceived difficulty in protective behavior, relevant parties need to optimize the external environment, making protective behavior simpler and more feasible. Scenic areas should improve infrastructure, such as adding garbage sorting points, clearly marked environmental protection signs, and providing convenient environmental protection manuals. These measures help tourists easily understand and implement protective measures. Utilizing technological means is also an effective approach. For example, developing dedicated environmental protection apps for scenic areas can provide tourists with real-time eco-friendly behavior guidance, demonstrating specific operations like correctly sorting garbage and protecting natural vegetation. This technical support not only enhances the operability of the behavior but also strengthens tourists’ confidence in the effectiveness of their protective actions.

#### Emphasize the guidance of subjective norms on group behavior

Although subjective norms have a relatively weaker influence on ecological protection behavior (*β* = 0.177), they still play an important role in shaping social expectations and group atmosphere. Shaping a positive social identity and group sense of responsibility can strengthen the sustainability of protective behavior through external pressure. Governments should utilize public service advertisements, public figure demonstrations, and reward programs to create a public opinion atmosphere that “protecting the ecology is a social responsibility.” For example, promoting cases of environmental behaviors by public figures can enhance tourists’ social identity. Moreover, cultural and tourism departments can design environmental protection point systems, encouraging tourists to participate in environmental actions by redeeming discounts or souvenirs with points. Scenic areas can set up volunteer leaderboards or host environmental protection competitions to form a group interaction and positive competition atmosphere.

### Theoretical significance

This study validates the applicability of the Theory of Planned Behavior in the context of ecological protection behaviors at intangible cultural heritage sites, extending its application in specific cultural backgrounds. The findings confirm that behavioral attitude, subjective norms, and perceived behavioral control significantly promote tourists’ ecological protection intentions, broadening the TPB’s research perspective in cultural contexts.

By incorporating personal norms into the TPB framework, the study reveals the unique role of moral responsibility in tourists’ behaviors. Tourists’ internalized moral values have a more significant influence on protective behavioral intentions compared to traditional TPB variables, enhancing the model’s explanatory power and underscoring the importance of integrating moral factors. From an environmental psychology perspective, the study explores the mechanisms influencing tourists’ ecological protection behaviors under cultural backgrounds. Unlike previous studies that focus on behavioral attitude or subjective norms, our research finds that tourists’ protective intentions are more driven by personal norms at culturally rich intangible heritage sites. The cultural background endows tourists with deeper emotional connections and moral responsibility, making them view ecological protection as an internal obligation. This supports environmental psychology’s core viewpoint that cultural connotations and situational characteristics play important roles in forming behavioral intentions. The study enriches the exploration of individual-environment interactions, indicating that ecological protection behavior is both a manifestation of social norms and an expression of psychological identification and moral responsibility. This offers important insights for motivating sustainable behaviors and supports policy design and practice for ecological protection at intangible cultural heritage sites.

### Practical significance

The findings provide a scientific basis for formulating ecological protection policies at intangible cultural heritage sites by demonstrating the significant impacts of personal norms, behavioral attitude, perceived behavioral control, and subjective norms on ecological protection intentions. Governments and management departments can design targeted protection strategies that enhance tourists’ internal value recognition, strengthen positive behavioral attitudes, and optimize external conditions.

The research highlights the core role of tourists’ internal moral sense of responsibility in ecological protection behaviors, reflecting that ecological protection is both an environmental governance demand and an important embodiment of cultural value continuation. Integrating cultural value into ecological protection actions promotes deep integration of culture and ecology. Cultural protection practices at intangible heritage sites can complement ecological protection needs, such as combining unique cultural projects with ecological education or promoting green tourism through traditional craft innovation. This integration enhances practical effectiveness and provides a path for coordinated development in environmental and cultural dimensions. Analyzing tourists’ behavioral attitudes and perceived behavioral control offers clear directions for scenic spots and tourism enterprises to motivate tourist participation. Providing intuitive environmental facilities, designing low-carbon travel projects, and developing participatory activities can enhance environmental awareness and behavioral execution. Although subjective norms play a relatively weaker role, their importance in shaping group behavior and social consensus is significant. Enhancing tourists’ social identity and group belonging can promote public support and participation in ecological protection. Governments and related agencies can strengthen publicity and guidance, including promoting environmental role models, organizing nationwide themed activities, and building interactive participation platforms, helping tourists recognize the social value of ecological protection and enhancing their sense of responsibility.

### Limitations and future research

This study has certain limitations. First, moderate multicollinearity exists among variables such as behavioral attitude, subjective norms, and perceived behavioral control (VIF values between 5.742 and 6.852), which may affect the independence of variable effects in the model. Although not severe, it may introduce bias in result interpretation. Second, the sample mainly comes from the Ancient City of Dali. Despite covering some non-local tourists through online questionnaires, the data focus on Dali’s cultural and ecological background, potentially limiting applicability in other heritage sites with significant cultural differences. Tourists’ behaviors are influenced by different cultural norms and values, necessitating further verification in broader contexts.

Future research can expand and optimize the study. Research scopes should cover more regions to reflect tourists’ behaviors in different cultural backgrounds, ecological features, and heritage types. Exploring cultural diversity’s impact on behavioral drivers will help comprehensively understand ecological protection behaviors. Research methods can be diversified. While quantitative research reveals relationships between variables, qualitative research can deeply analyze complex motivations. Methods like interviews or focus groups can help understand the formation of internal moral norms and their manifestations.

Additionally, future studies can explore mediating and moderating variables in behavioral intentions. Additionally, future studies can explore mediating and moderating variables in behavioral intentions. The TPB points out that behavioral beliefs are influenced by the joint effect of social culture and cognitive factors (Ajzen, 1991). The relationship between personal norms and altruistic behavior is not a single internal drive but a result of the interaction between social cultural background and cognitive factors (Schwartz, 1977). Exploring these complex relationships, especially differences in acceptance and expression of behavioral norms under different cultural backgrounds, will reveal the multidimensional drivers of ecological protection behaviors more comprehensively.

## Data Availability

The original contributions presented in the study are included in the article/Supplementary material, further inquiries can be directed to the corresponding author.
